# Monolayer SnI_2_: An Excellent p-Type Thermoelectric Material with Ultralow Lattice Thermal Conductivity

**DOI:** 10.3390/ma15093147

**Published:** 2022-04-26

**Authors:** Qing-Yu Xie, Peng-Fei Liu, Jiang-Jiang Ma, Fang-Guang Kuang, Kai-Wang Zhang, Bao-Tian Wang

**Affiliations:** 1 Institute of High Energy Physics, Chinese Academy of Sciences (CAS), Beijing 100049, China; qyxie@ihep.ac.cn (Q.-Y.X.); pfliu@ihep.ac.cn (P.-F.L.); majj88@ihep.ac.cn (J.-J.M.); 2 School of Physics and Optoelectronics, Xiangtan University, Xiangtan 411105, China; 3 Spallation Neutron Source Science Center (SNSSC), Dongguan 523803, China; 4 School of Physics and Electronic Information, Gannan Normal University, Ganzhou 341000, China; kuangfg1987@126.com; 5 Collaborative Innovation Center of Extreme Optics, Shanxi University, Taiyuan 030006, China

**Keywords:** thermoelectrics, electronic transport, thermal transport

## Abstract

Using density functional theory and semiclassical Boltzmann transport equation, the lattice thermal conductivity and electronic transport performance of monolayer SnI_2_ were systematically investigated. The results show that its room temperature lattice thermal conductivities along the zigzag and armchair directions are as low as 0.33 and 0.19 W/mK, respectively. This is attributed to the strong anharmonicity, softened acoustic modes, and weak bonding interactions. Such values of the lattice thermal conductivity are lower than those of other famous two-dimensional thermoelectric materials such as MoO_3_, SnSe, and KAgSe. The two quasi-degenerate band valleys for the valence band maximum make it a p-type thermoelectric material. Due to its ultralow lattice thermal conductivities, coupled with an ultrahigh Seebeck coefficient, monolayer SnI_2_ possesses an ultrahigh figure of merits at 800 K, approaching 4.01 and 3.34 along the armchair and zigzag directions, respectively. The results indicate that monolayer SnI_2_ is a promising low-dimensional thermoelectric system, and would stimulate further theoretical and experimental investigations of metal halides as thermoelectric materials.

## 1. Introduction

With more than 60% of energy in the world lost in the form of waste heat, the thermoelectric system has attracted widespread attention, since it directly converts the waste heat to electric energy through the Seebeck effect. It has the advantages of small size, high reliability, no pollutants, and a feasibility in a wide temperature range. Such a system is widely used in aerospace exploration and industrial production, such as in space probes, thermoelectric generators, and precise temperature controls [1,2]. The converting efficiency of TE materials is ruled by the dimensionless figure of merit (ZT); ZT = *S*^2^*σT*/(*κ*_L_+ *κ*_e_), where *S*, *σ*, *T*, *κ*_L,_ and *κ*_e_ are the Seebeck coefficient, electrical conductivity, absolute temperature, lattice thermal conductivity, and electronic thermal conductivity, respectively. The electronic transport properties *S*, *σ*, and *κ*_e_, have a complex coupling relationship, and are difficult to decouple, even though they can be modified via carrier concentration [3,4,5]. Historically, two aspects were established to enhance ZT: one aspect is to optimize carrier concentration by the band structure, engineered to enhance the power factor (*σS*^2^) [6,7,8,9], and the other aspect is to reduce the lattice thermal conductivity, via alloying and nanostructuring [10]. Alternatively, it is more attractive to seek materials with an intrinsic low lattice thermal conductivity (generally associated with complex crystal structures [11]), strong anharmonicity [12,13], lone pair electrons [14,15], and liquid-like behavior [16,17,18], etc.

The group IVA metal dihalides are candidates for semiconductor optical devices and perovskite solar cells, due to their excellent properties, such as the visible-range band gap and the thickness-dependent band structure [19,20,21,22]. However, the application of the Pb-based materials, such as the layered 2H-PbI_2_, has been greatly limited by their toxicity and environmental unfriendliness [23,24,25]. In addition, the surface of the bulk SnI_2_ is generally very rough and accompanied by many defects, which strongly scatters carriers [26]. Fortunately, its vdW monolayer has been experimentally realized, via molecular beam epitaxy [27]. The low dimensionality provides an effective conductive channel for carriers, and also suppresses phonon thermal transport [28]. Thus, monolayer SnI_2_ is a preferable option over bulk SnI_2_ and monolayer PbI_2_ in use as a TE material, and deserves to be carefully explored in a systematic study. 

In this work, combining first-principles calculations and the semiclassical Boltzmann transport equation, the TE properties of monolayer SnI_2_ were systematically explored. Results show that the intrinsic ultralow lattice thermal conductivity originates from the strong anharmonicity, weak bonding, and softened acoustic modes. The Grüneisen parameter, phonons scattering phase space, and phonon relaxation time were calculated to understand the micro-mechanism of the phonon transports. The two quasi-degenerate band valleys for the valence band maximum (VBM) in its electronic band structure led to a p-type TE material. The maximum ZT value along the armchair and zigzag directions at 800 K reach 4.01 and 3.34, respectively, using optimal p-type doping. These results indicate that monolayer SnI_2_ exhibits an extraordinary TE response, and is an ideal material for TE applications.

## 2. Computational Methods

The first-principles calculations were implemented in the Vienna ab initio simulation package (VASP, VASP.5.3, Wien, Austria) [29]. The generalized gradient approximation (GGA) [30,31] in the Perdew–Burke–Ernzerhof (PBE) [32] form was employed to deal with the exchange–correlation functional, with a cutoff of 300 eV on a 9 × 9 × 1 Monkhorst–Pack *k*-mesh. To screen the interactions between adjacent images, the length of the unit cell of 20 Å was used along the z direction. The geometry structure was fully relaxed, with a criterion of convergence for residual forces of 0.001 eV/Å, and the total energy difference converged to within 10^−8^ eV/Å. To obtain an accurate band gap and electronic transport performance, the Heyd–Suseria–Ernzerhof (HSE06) [33] method was employed.

The electronic transport properties of the monolayer SnI_2_ were calculated by solving the semiclassical Boltzmann transport equation, utilizing the BoltzTraP code (Georg K. H. Madsen, Århus, Denmark) [34] with a dense 35 × 35 × 1 *k*-mesh. The constant relaxation time approach (CRTA) was used, since the relaxation time is not strongly dependent on the energy scale of k_B_T, and has accurately predicted TE properties of multitudinous materials. In this work, the electrons relaxation time was calculated using the deformation potential (DP) theory [35], which considers the primarily acoustic phonon scatterings, but ignores effects of the optical phonons in the single parabolic band (SPB) model. Based on the rigid band approximation (RBA) [36] and CRTA, the transport coefficients *S*, *σ*, and *κ*_e_ can be obtained by:

(1) $$S=\;\frac{ek_{B}}{\sigma }\int \Xi (\varepsilon )(-\frac{\partial f_{0}}{\partial \varepsilon })\frac{\varepsilon -u}{k_{B}T}d\varepsilon$$

(2) $$\sigma =e^{2}\int \Xi (\varepsilon )(-\frac{\partial f_{0}}{\partial \varepsilon })\frac{\varepsilon -\mu }{k_{B}T}d\varepsilon$$

(3) $$\kappa_{e}=\kappa_{0}-\sigma S^{2}T=L\sigma T$$

(4) $$\Xi =\sum \nu_{k}\nu_{k}\tau_{k}$$

where *k_B_*, *f*_0_, and Ξ(ε) are the Boltzmann constant, the Fermi–Dirac distribution function, and the transport distribution function, respectively. The calculation of the relaxation time τ was extremely difficult, due to the various complex scattering mechanism in the crystals. The relaxation time was calculated based on the DP theory in the SPB model, which considers the predominant scatterings between carriers and acoustic phonons in the low-energy region. In fact, the scattering matrix element (${\left|M(\vec{k},\vec{k’})\right|}^{2}$) of the acoustic phonons in the long-wavelength can be approximated as *k*_B_*TE*_1_*^2^/C_ii_*, where *C*_ii_ and *E*_1_ are the elastic and DP constants, respectively. Thus, for the 2D material, the carrier mobility and electrons relaxation time can be approached as [37,38]

(5) $$\mu_{2D}=\frac{eh^{3}C_{ii}}{8\pi^{3}k_{B}Tm_{d}m*E_{1}{}^{2}}$$

(6) $$\tau =\frac{um*}{e}$$

where *h*, m*, and *m_d_* are the Planck constant, the effective mass along the transport direction, and the averaged effective mass, respectively. 

The harmonic second-order interaction force constants (2nd IFCs) and phonon spectrum were calculated using the Phonopy package [39], using a 2 × 2 × 1 supercell with a 5 × 5 × 1 *k*-mesh using the finite-difference method [40]. The anharmonic third-order interaction force constants (3rd IFCs), which consider the interactions between the sixth-nearest-neighbor atoms, were obtained using the ShengBTE package (ShengBTE version 1.0.2, Wu Li, Grenoble, France; Phonopy version 2.11.0, Atsushi Togo, Sakyo, Japan) [41] with the same supercell. The lattice thermal conductivity and phonon transport properties were obtained using the self-consistent iterative solution of the Boltzmann transport equation, with a dense 36 × 36 × 1 mesh, which had a good convergence. Based on the Boltzmann transport equation, with the Fourier’s low of heat conduction, the matrix elements of the phonon thermal conductivity can be expressed by [42,43]

(7) $$\kappa_{p.\alpha \beta }=\frac{1}{N_{q}}\sum_{\lambda }c_{\lambda }v_{\lambda ,\alpha }v_{\lambda ,\beta }\tau_{\lambda }$$

where *α* and *β* are the Cartesian indices, *N_r_* is the total number of q-points sampled in the first Brillouin zone, and *c_λ_*, *v_λ_,* and *τ_λ_* are the mode-specific heat capacities, phonon group velocity, and the relaxation time, respectively. Here, the phonon thermal properties were calculated based on the 3rd IFCs, ignoring the fourth- and higher-order terms. This strategy described the phonon behavior of the most anharmonic materials [44,45]. To define the effective thickness of two-dimensional (2D) materials, the summation of interlayer distance and the vdW radii of the outermost surface atoms was adopted [44]. 

## 3. Results and Discussion

### 3.1. Geometry and Electronic Structure

**Figure 1:**
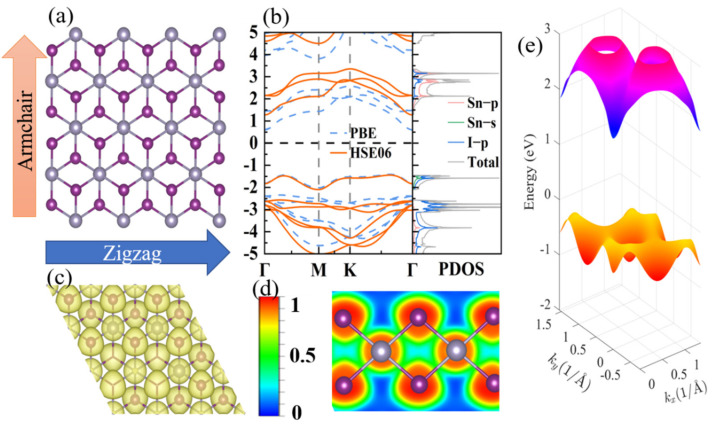
(**a**) Top view of the atomic structure of SnI_2_ monolayer. (**b**) The electronic band structures calculated with PBE and HSE06 hybrid functional potentials. (**c**,**d**) the 3D and 2D ELF maps. (**e**) The 3D electronic band structure calculated with PBE.

Monolayer SnI_2_ crystallizes in a hexagonal lattice with space group P-3m1 (164), as shown in Figure 1. The optimized lattice parameter is 4.57 Å, which is in good agreement with the previous experimental data of 4.48 Å [27]. The structure is analogous to H-MoS_2_ [46], consisting of three layers, with Sn atoms as the middle layer, and I atoms as the upper and lower atomic layers. The electron localization function (ELF) provides a deeper insight to characterize the nature of, and strengthen, the chemical band. Figure 1c shows the calculated three-dimensional (3D) ELF map (isosurface level of 0.97). The ELF around I is in the shape of a “mushroom”, suggesting the existence of the lone pair electrons. Moreover, the electron sharing is better visualized by the 2D ELF map in Figure 1d. The interstitial electrons between Sn and I are close to I atoms, and the value of the area between them is 0.5, which shows the characteristics of a free electron gas and indicates a weak bonding between Sn and I atoms. The electronic repulsion between the lone pair electrons and the Sn–I bonding electrons results in strong anharmonicity, such as in CuSbS_2_ [14]. The complex structure and lone pair electrons are also beneficial to its low lattice thermal conductivity.

The electronic structure plays a crucial role in characterizing the electronic transport properties. As shown in Figure 1b, the electronic band structures obtained from the PBE and HSE06 hybrid function potentials are presented. They exhibit analogous band structures, except for a more accurate bandgap (2.71 eV) that approaches the experiment value (2.9 eV) [27] for the latter structure. The conduction band mainly originates from the *p* orbitals of the Sn atom and the *p* orbitals of the I atom, whereas the valence band consists of the *s* orbital of the Sn atom and the *p* orbitals of the I atom. Below the VBM, there are two quasi-degenerate band valleys along the Γ–M and Γ–K directions with an energy difference of ~0.03 eV, which is far less than those in SnTe (~0.35eV) [47] and PbTe (~0.15 eV) [1]. The multi-energy valley enhances the TE performance and is verified in many materials [48,49]. However, the behavior of band valleys degenerate do not exist for the conduction band minimum (CBM), which is located at the Γ point. Hence, it is expected that the TE performance of the p-type could be superior to that of the n-type.

### 3.2. Electronic Transport Properties

**Figure 2:**
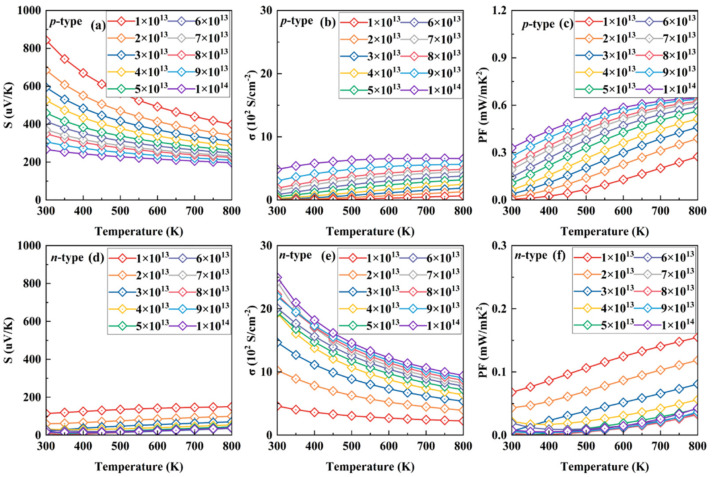
Seebeck coefficient, electronic conductivity, and PF as a function of temperature under various concentrations of p- (**a**–**c**) and n-type (**d**–**f**) doping.

All the parameters for electronic transport, calculated according to the DP theory, are tabulated in Table 1. Based on the reasonable relaxation time, all the electronic transport coefficients, Seebeck coefficient *S*, electrical conductivity *σ*, and power factor (PF), as a function of temperature at the corresponding optimal carrier concentration for p-type SnI_2,_ range from 1 × 10^13^ to 1 × 10^14^ cm^−2^, and are presented in Figure 2a–c. For comparison, those for n-type SnI_2_ at the same condition are also plotted in Figure 2d–f. Here, the tiny anisotropy characteristics are ignored. 

Increasing the carrier concentration, the chemical potential enters in the deeper energy levels for both p- and n-types of SnI_2_, resulting in a decreased Seebeck coefficient at the same temperature. It is clear that the values under p-type doping are always far larger than those under n-type at the same condition, which can be attributed to the multi-band character near the VBM, as shown in Figure 1b. For example, the absolute values of the *S* under p- and n-types of doping at 700 K are 439 and 146 uV/K, respectively, for a doping concentration of 1 × 10^13^ cm^−2^. In addition, the trend of *S* under p-type doping is opposite to that under n-type. The former decreases while the latter increases upon heating. 

Compared with *S*, the electronic conductivity σ exhibits a different behavior, due to the complete relationship between them, as Equations (1) and (2) show. Interestingly, in all cases, the electrical conductivities for p-type SnI_2_ are always far less than those for n-type. For example, the values under p- and n-type doping are 0.46 and 2.43 S/cm^−2^, respectively, at 700 K for a doping concentration of 1 × 10^13^ cm^−2^. At 800 K, the electrical conductivity under p-type (n-type) increases from ~0.67 S/cm^−2^ (~2.24 S/cm^−2^) for 1 × 10^13^ cm^−2^ to 6.35 S/cm^−2^ (9.43 S/cm^−2^) for 1 × 10^14^ cm^−2^.

Ultimately, the PF decouples the complete relationship between the Seebeck coefficient and electrical conductivity. It is clearly seen that the p-type SnI_2_ possesses significantly higher PF values than those of the n-type system. This is in good agreement with the previous analysis for the electronic structure. At 800 K, the PF for p-type increases from ~0.27 mW/mK^2^ for 1 × 10^13^ cm^−2^ to 0.62 mW/mK^2^ for 1 × 10^14^ cm^−2^. For the n-type system, however, the PF decreases from ~0.15 mW/mK^2^ for 1 × 10^13^ cm^−2^ to 0.04 mW/mK^2^ for 1 × 10^14^ cm^−2^. Thus, it is expected that the p-type SnI_2_ possesses a more excellent performance than that of the n-type. According to the Wiedemann–Franz law, *κ*_e_ = L*σT*, where L is the Lorenz number, the classical value L = (π*k*_B_)^2^/3e^2^ ≈ 2.44 × 10^−8^ WΩK^−2^ is adopted. This value meets the result of *κ_0_-σS^2^T* [50]. Thus, the electronic conductivity is proportional to the electronic thermal conductivity. The results in the present work also obey this rule.

### 3.3. Phonon Transport Properties

**Figure 3:**
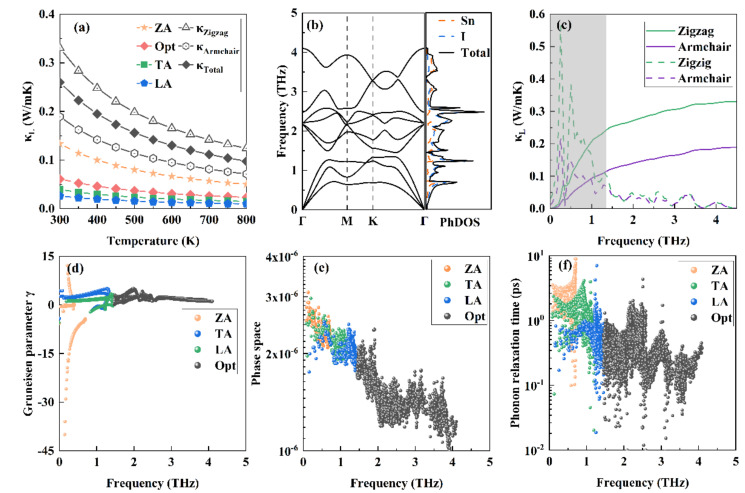
(**a**) Calculated lattice thermal conductivity. The contributions from the ZA, TA, LA, and optical modes to the total κ_L,_ as well as the lattice thermal conductivities along the armchair and the zigzag directions, are shown. (**b**) Phonon dispersion curves and corresponding PhDOS. (**c**) Cumulative thermal conductivity and the derivatives (dashed line) with respect to frequency. (**d**–**f**) Grüneisen parameter γ, phonon scattering phase space, and phonon relaxation time of the ZA, TA, LA, and optical modes.

As shown in Figure 3a, the calculated total *κ*_L_ is very low in the wide temperature of 300–800 K. Remarkably, the low room temperature phonon thermal conductivities of 0.33 and 0.19 W/mK along the armchair and zigzag directions, respectively, are fundamentally lower than those previously reported values for 2D MO_3_ (1.57 W/mk) [51], SnSe (2.77 W/mK) [13], and CaP_3_ (0.65 W/mK) [52]. Through analyzing the contributions of the acoustic phonon branches along the in-plane (TA and LA) or out-of-plane (ZA), as well as the optical phonon modes to the total *κ*_L_, results show that the main contribution to the *κ*_L_ is from the ZA mode. In the following, we reveal the origins of such ultralow phonon conductivity, and present a comprehensive analysis to support this result.

The phonon dispersion curves are presented in Figure 3b. With three atoms in its primitive cell, there are three acoustic and six optical phonon modes for monolayer SnI_2_. It is dynamically stable, since no imaginary frequency is observed. Near the long wave limit, the TA and LA branches are in linear trend, whereas the ZA branch exhibits a quadratic trend. These features are typical for 2D materials, and can be explained with the elastic theory of thin plate [53]. It is clearly seen that a narrow phonon gap of about 0.1 THz separates the phonon modes into the acoustic phonon part (0~1.35 THz) and the optical phonon part (1.45~4.10 THz). The cutoff acoustic phonon frequency, which is as low as 1.35 THz, is lower than those for SnSe (1.6 THz) and SnS (1.9 THz) [13]. The low-lying acoustic modes, as well as the soft mode for TA near the M point, imply the weak bonding between Sn and I atoms, consistent with the previous analysis. From the corresponding phonon density of states (PhDOS), the contributions from the I atomic vibrations are apparently larger than those from Sn, within the region of 2.8 to 4.10 THz. Both Sn and I evidently contribute in a wide energy range, implying the nature of covalent bonding [12]. In addition, it is recalled that the decoupling of the in-plane and out-of-plane phonon modes results in ultrahigh phonon thermal conductivity for graphene, due to its one atom plane nature [53,54]. However, such full decoupling behavior should not be observed for a finite thickness 2D system, such as the case of the present studied monolayer SnI_2_. 

To explain the anomalous thermal transport behavior of monolayer SnI_2_, the cumulative lattice conductivity, and their derivatives, with respect to frequency at 300 K are calculated and presented in Figure 3c. Clearly, the *κ*_L_ is mainly caused by phonons of the cutoff acoustic part (the shadow region < 1.35 THz). Specifically, the contributions from ZA, TA, LA, and the optical branches are 51.21%, 15.14%, 10%, and 23.54%, respectively. For graphene, the low frequency (0–5 THz) also dominates the *κ*_L_ and the ZA mode, and contributes 75% to *κ*_L_ [54]. Comparing with graphene, the structure of the monolayer SnI_2_ lacks mirror symmetry. Thus, the mirror symmetry does not reflect whether the ZA mode dominates the thermal transport [55].

Generally, there are two factors that dominate the κ_L_: (i) anharmonic interaction matrix elements, and (ii) the inverse of phonon phase space volume. The Grüneisen parameter γ is generally employed to quantify the strength of anharmonicity [41]. Figure 3d shows the calculated γ of the ZA, TA, LA, and optical modes of the SnI_2_ monolayer. As a large |γ| implies strong anharmonicity, it can be seen that the ZA mode exhibits giant anharmonicity. The large |γ| of this out-of-plane mode means that the anharmonicity of the bonding between Sn and I atoms in the vertical direction of the monolayer plate is strong. The phonon scattering phase space reveals all available scattering processes, which are ruled by the energy and (quasi)momentum conservation. As shown in Figure 3e, the total scatting phase space of the ZA, TA, and LA modes are 1.55 × 10^−3^, 1.41 × 10^−3^, and 1.35 × 10^−3^, respectively, which confirms more abundant scattering channels of the ZA mode, than those of the TA and LA modes. The larger the phonon phase space, the greater the contribution to the *κ*_L_, thus, validating the decreasing contributions to *κ*_L_ from the ZA, TA, and then LA modes. The phonon relaxation time provides a deeper microcosmic insight to understand the ultralow *κ*_L_ of SnI_2_. Compared with the monolayer SnP_3_ [38], monolayer SnI_2_ possesses a shorter phonon relaxation time, implying an ultralow *κ*_L_.

### 3.4. Thermoelectric Figure of Merit

**Figure 4:**
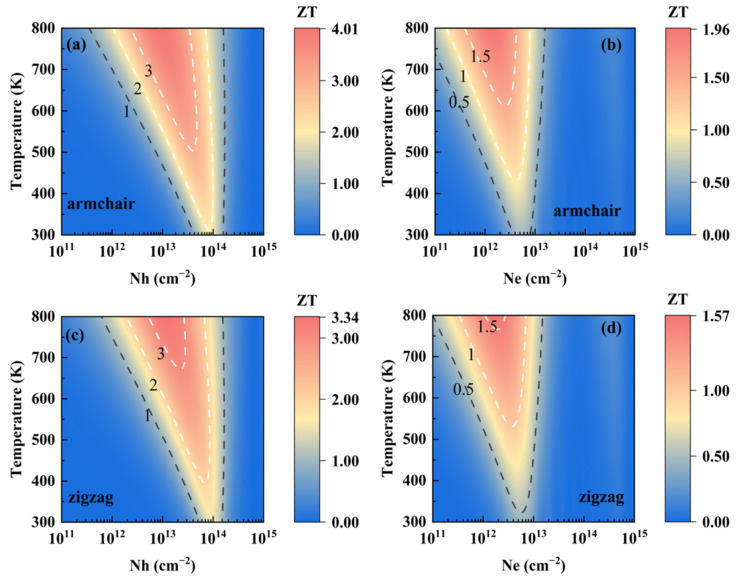
Contour maps of ZT as functions of both temperature and carrier concentration for monolayer SnI_2_: (**a**) p-type and (**b**) n-type along the armchair direction, (**c**) p-type and (**d**) n-type along the zigzag direction.

By combining the phonon and electron transport coefficients, the ZT of SnI_2_ under p- and n-types of doping as functions of temperature and carrier concentration are presented in Figure 4. Owing to the calculated thermal conductivity, along the zigzag direction is slightly larger than the armchair direction, and it is expected that the ZT along the armchair direction is higher than that along the zigzag direction. In addition, the p-type SnI_2_ is obviously superior to the n-type, since the two quasi-degenerate band valleys in VBM leads to a larger Seebeck coefficient. Results show that monolayer SnI_2_ is thermally stable up to 800 K, by performing ab initio molecular dynamic (AIMD) simulations (see Figure S1). The values of ZT are as high as 4.01 and 3.34 along the armchair and zigzag directions, respectively, around concentrations of 1.0 × 10^13^ and 1.2 × 10^13^ cm^−2^, at 800 K. Such large values are better than the experimental value of 2.6 for the well-known TE material SnSe along the specific axis at 925 K [3]. In addition, the doping carrier concentrations at such levels have been realized experimentally in monolayer MoS_2_ [56]. Therefore, such a high TE value for monolayer SnI_2_ is possible, and indicates its excellent potential TE performance. 

To clearly compare the TE performances of the monolayer SnI_2_ with some famous TE materials, the *κ*_L_ and the max ZT are listed in Table 2. According to these results, the monolayer SnI_2_ is an emerging candidate for TE devices, due to its ultrahigh ZT. As seen, the *κ*_L_ of monolayer SnI_2_ is very low, almost a twentieth of the monolayer SnP_3_ [38], and is greatly lower than those of tin selenide and tin sulfide [13,48]. Such low value of the *κ*_L_ for monolayer SnI_2_ make it a typical 2D TE system. After all, the electronic transport properties can be easily modulated in experiments, while the lattice thermal conductivity is very difficult to change. In addition, the *κ*_L_ values at the level of 0.1-0.5 W/mK in experiments are very few. To the best of our knowledge, the complex systems of CsAg_5_Te_3_ (0.18 W/mK) [57] and CsCu_5_Se_3_ (0.4~0.8 W/mK) [58], as well as the bulk superlattice material Bi_4_O_4_SeCl_2_ (0.1 W/mK) [59], are typical low *κ*_L_ TE materials. Overall, the low thermal conductivity, as well as the high ZT, make monolayer SnI_2_ a good TE material for low-dimensional devices. 

## 4. Conclusions

In summary, the TE properties of monolayer SnI_2_ were studied using DFT and the semi-classical Boltzmann transport equation. The results indicate that this 2D material possesses intrinsically ultralow lattice thermal conductivity. Its strong anharmonicity, weak bonding, and softened acoustic branches greatly suppress the phonon transport, and result in an ultralow *κ*_L_ of 0.33 and 0.18 W/mK at 300 K along the zigzag and armchair directions, respectively. The p-type SnI_2_ possesses a superior electric transport performance than the n-type one, due to the two quasi-degenerate band valleys in its VBM. The ZT at 800 K under p-type doping is as high as 4.01 along the armchair direction. Collectively, these results indicate the great advantages of monolayer SnI_2_ for converting heat energy with high efficiency at high temperatures. Generally, when the ZT value of a material exceeds 1.0, it is considered as an ideal TE material. Therefore, the high ZT of monolayer SnI_2_ demonstrates it as an emerging candidate for TE applications. 

## Figures and Tables

**Table 1:** The calculated DP constant E_1_, elastic constant, effective mass, carrier mobility, and relaxation time along the zigzag and armchair directions in monolayer SnI_2_ at 300 K.

Direction	Type	E_1_ (eV)	C_ii_ (J/m^2^)	m* (*m*_0_)	*u* (cm^−2^V^−1^S^−1^))	*τ* (fs)
Zigzag	e	−4.44	17.92	0.59	42.52	17.41
	h	−4.43	17.92	0.73	40.16	16.72
Armchair	e	−4.25	17.42	0.84	30.48	15.17
	h	−4.49	17.42	0.68	35.14	13.60

**Table 2:** *κ*_L_ at 300 K and the max ZT at corresponding maximum thermodynamic temperature for monolayer SnI_2,_ as well as some typical TE materials.

Material	*κ*_L_ (W/mK)	ZT	
SnI_2_	0.26	4.01 (800 K)	This work
SnS	1.5	1.00 (750 K)	Ref. [13]
SnSe	0.6	1.50 (750 K)	Ref. [13]
SnP3	4.97	3.46 (500 K)	Ref. [38]
SnSe	1.12	0.85 (900 K)	Ref. [48]
LaCuOSe	0.84	2.71 (900 K)	Ref. [50]

## Data Availability

The data presented in this study are available on request from the corresponding authors. The data are not publicly available due to ongoing research in the project.

## Supplementary Materials

The following supporting information can be downloaded at: https://www.mdpi.com/article/10.3390/ma15093147/s1, Figure S1: Free energy fluctuations with respect to time and equilibrium structures of SnI2 monolayer by AIMD simulations at 800 K.
