# Supplementary material for: Monolayer SnI2: An Excellent p-Type Thermoelectric Material with Ultralow Lattice Thermal Conductivity
Source: Materials (Basel). 2022 Apr 26;15(9):3147. doi: 10.3390/ma15093147 (PMC9101867; doi:10.3390/ma15093147)
Supplement: Supplementary file 1 [file materials-15-03147-s001.zip › materials-1664144-supplementary.pdf]

Supporting Information

Monolayer SnI_2: an excellent p-type thermoelectric material with ultralow lattice thermal conductivity

Qing-Yu Xie[a, b], Jiang-Jiang Ma[a, c], Peng-Fei Liu[a, c], Fang-Guang Kuang[d], Kai-wang Zhang*[a] and Bao-Tian Wang*[a, c, e]

[a] Institute of High Energy Physics, Chinese Academy of Science (CAS), Beijing 100049, China. E-mail: wangbt@ihep.cn

[b] School of Physics and Optoelectronics, Xiangtan University, Hunan 411105, China. E-mail: kw@xtu.edu.cn

[c] Spallation Neutron Source Science Center (SNSSC), Dongguan 523803, China

[d] School of Physics and Electronic Information, Gannan Normal University, Ganzhou 341000, China

[e] Collaborative Innovation Center of Extreme Optics, Shanxi University, Taiyuan 030006, China

E-mail: wangbt@ihep.cn

The Thermal Stability

The thermodynamic stability of the 2D system is of great importance. We run the ab-initio molecular dynamics (AIMD) simulations at 800 K for SnI_2 monolayers. Results show that SnI_2 monolayer can be maintained up to 10 ps at 800 K, suggesting they are stable at high temperature.

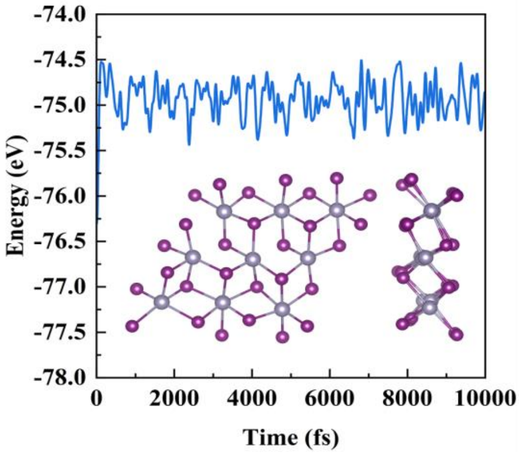

Figure S1. Free energy fluctuations with respect to time and equilibrium structures of SnI_2 monolayer by AIMD simulations at 800 K.
